# Osteonecrosis in Youth with Sickle Cell Disease—A Narrative Review

**DOI:** 10.3390/children13091143

**Published:** 2026-08-26

**Authors:** Melissa Fiscaletti

**Affiliations:** Azrieli Research Centre of CHU Sainte Justine, Department of Pediatrics, University of Montreal, Montreal, QC H3T 1J4, Canada; melissa.fiscaletti@umontreal.ca

**Keywords:** sickle cell disease, osteonecrosis, avascular necrosis, bone infarcts, children

## Abstract

**Highlights:**

**What are the main findings?**

- Magnetic resonance imaging is the most sensitive tool for early diagnosis and plays a central role in disease staging and risk stratification.
- Management requires a multidisciplinary approach that includes analgesia, physical therapy, activity modification, optimization of bone health, and treatment of associated endocrine abnormalities.
- Despite growing recognition of osteonecrosis as a major complication of SCD, important gaps remain in our understanding of disease progression and optimal treatment.

**What is the implication of the main finding?**

- Earlier identification of high-risk patients and the development of effective joint-preserving strategies remain key priorities for future research.

**Abstract:**

Osteonecrosis can cause significant and chronic musculoskeletal morbidity in children and adolescents with sickle cell disease (SCD). Although the femoral head is most frequently affected, lesions can also occur in the humeral head, vertebral bodies, knees, and other joints. Progressive joint damage can result in chronic pain, impaired mobility, loss of function, and the need for surgical intervention at a young age. As survival continues to improve in SCD, the long-term burden of osteonecrosis is becoming increasingly important. This narrative review summarizes the current understanding of osteonecrosis in pediatric and adolescent SCD. We review its epidemiology, pathophysiology, clinical presentation, imaging findings, and management. Disease burden increases with age and is closely linked to markers of severe SCD, including frequent vaso-occlusive crises and acute chest syndrome.

## 1. Epidemiology, Nomenclature and Natural History

Sickle cell disease (SCD) is an inherited hemoglobinopathy characterized by acute and chronic complications across multiple organs. Despite major advances in screening, supportive care and disease-modifying therapies, SCD remains a major public health challenge in pediatric care, affecting nearly 8 million people worldwide [1]. Youth with mutations in the β-globin gene experience vaso-occlusive episodes and can cause severe pain and organ damage. Several genotypes are recognized, including HbSS, the most common form (87%), as well as HbSC and HbSβ-thalassemia [2].

The skeleton is particularly susceptible to the effects of repeated ischemic injury. Among these complications, osteonecrosis, also known as avascular necrosis or ischemic necrosis, is one of the most frequent and morbid manifestations of SCD. The terms are interchangeable as they describe the same entity of bone death due to compromised blood supply. However, osteonecrosis is a more accurate term because the pathogenesis is not exclusively vascular but rather multifactorial. Furthermore, some cases exhibit revascularization and repair of the lesion [3,4]. A distinction should be made based on the anatomical location of the lesion. Osteonecrosis or avascular necrosis is the preferred term when the joint surface (epiphysis) is affected, while bone infarcts should be used to describe lesions in the metaphysis or diaphysis of the bone.

Osteonecrosis can affect multiple sites (Table 1), but most commonly affects the femoral head (ONFH), where it can progress to structural collapse, chronic pain and functional impairment. ONFH can occur in both children and adults and is a major cause of pain, disability, and impaired quality of life. Approximately 28% of children with SCD who develop ONFH experience a significant impact on their quality of life, physical function, and school participation [5]. Furthermore, nearly one quarter of affected adults require total hip replacement surgery by their third decade of life [6]. Other commonly affected locations are the humeral head [7,8], and vertebral bodies [7,9]. Lesions can also occur at the knees and elbows, although these sites are often less symptomatic [10].

The burden of disease increases with age. Early landmark studies from the Cooperative Study of Sickle Cell Disease following over 2000 individuals across their lifespan found evidence of osteonecrosis in about 10% of patients at baseline, with new cases continuing to develop during follow-up [11]. Although ON can be seen in children as young as 5 years of age [8,11], the prevalence increases during adolescence and early adulthood [11].

Recent studies suggest that the true burden of osteonecrosis may be higher than previously appreciated. Estimates vary depending on the population studied and the imaging method used. MRI can detect early and asymptomatic disease that may not be visible on plain radiographs [12]. In a large population-based study from California, 22% of over 6200 individuals with SCD developed ONFH, with a median age at diagnosis of 27 years [6]. In a Tunisian cohort consisting of 510 youth, the cumulative incidence increased from 2.3% at 10 years of age to 18.3% at 20 years and 33.6% at 30 years [13]. MRI-based studies in children have reported even higher rates, highlighting the frequency of subclinical disease and the limitations of radiographic screening alone. Many early lesions can be occult and asymptomatic. In a cross-sectional study of 42 children with a median age of 11 years, Voi et al. showed that when MRI is used to detect ONFH, the prevalence increases from 9.8% to 26% in children [11,12]. In adults, MRI-detected ONFH reaches almost 50% [12].

The risk of osteonecrosis is also closely linked to the severity of SCD. Frequent vaso-occlusive crises and a history of acute chest syndrome [6] have consistently been associated with a higher risk of developing osteonecrosis. Notably, the cumulative incidence of ONFH at 20 years was significantly higher among patients who experienced more than three vaso-occlusive crises per year compared with those who had fewer episodes (43.0% vs. 18.9%) [10,13]. Several studies have also identified associations with higher hemoglobin-to-hematocrit ratio [14], co-inherited α-thalassemia, particularly in patients with HbSS [15] disease and delayed initiation of disease-modifying therapy such as hydroxyurea [13]. Taken together, these findings support the view that osteonecrosis is a cumulative complication of ongoing vaso-occlusion and ischemic injury that becomes more common as patients age and disease severity increases.

## 2. Pathophysiology

**Figure 1:**
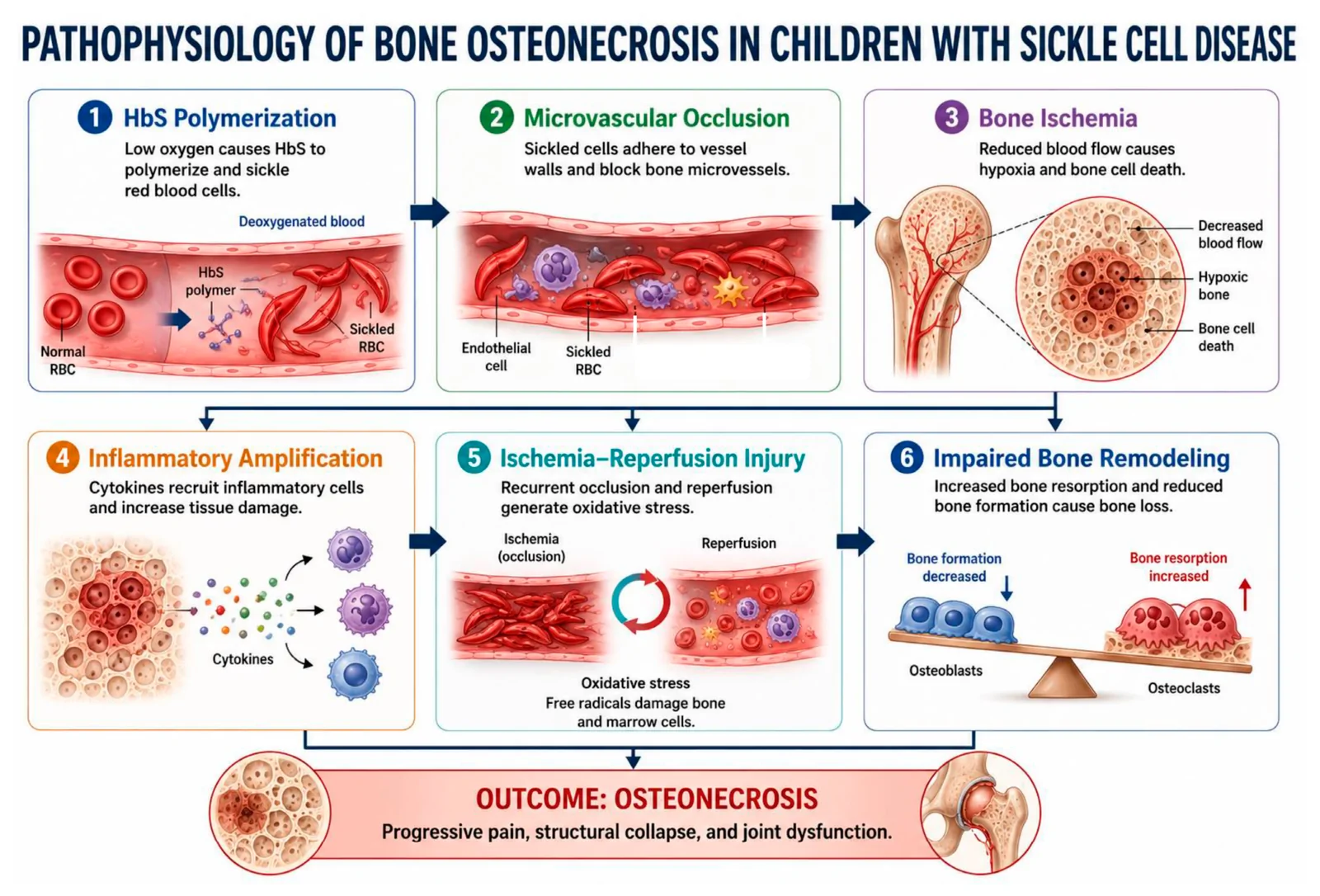
The pathophysiology of osteonecrosis in children with sickle cell disease is a multi-step process. This image was created with Canva.com.

Figure 1 summarizes the current understanding of osteonecrosis in SCD. Sickled erythrocytes, together with activated leukocytes and platelets, adhere to the vascular endothelium by increased expression of adhesion molecules. This promotes microvascular obstruction, reduces blood flow, and leads to recurrent episodes of ischemia within the bone and bone marrow. Repeated vaso-occlusion is further amplified by chronic endothelial dysfunction, inflammation, oxidative stress, and ischemia–reperfusion injury.

The location of the ischemic injury largely determines its long-term consequences. Infarction within the diaphysis or metaphysis of long bones, although painful, often heals through remodeling with limited structural damage. In contrast, when infarction occurs at the distal portion of the bones near the articular surfaces, long-term damage can occur [16,17]. The femoral head is particularly vulnerable because its blood supply is largely dependent on the lateral and medial femoral circumflex arteries and contains relatively limited collateral circulation. As a result, even brief interruptions in perfusion may lead to ischemic injury of the subchondral bone and marrow. Similar mechanisms contribute to osteonecrosis of the humeral head and other weight-bearing joints.

The necrotic bone will trigger an inflammatory cascade with cytokines, neutrophils further increasing endothelial dysfunction creating a vicious cycle of ischemia–reperfusion injury (Figure 1) [12]. Finally, a bone remodeling imbalance occurs with the hypoxia inducing osteoclastic activity (bone resorption) in greater proportion than osteoblastic activity (bone formation). The net result is necrosis with subchondral bone collapse.

## 3. Pediatric Clinical Presentation

The American Academy of Pediatrics recommends routine assessment for symptoms of osteonecrosis at the hip and shoulder beginning at between 5 and 12 years of age through history and physical exam. Education of patients and families of the risk and early manifestations of osteonecrosis should begin much earlier [18].

**Figure 2:**
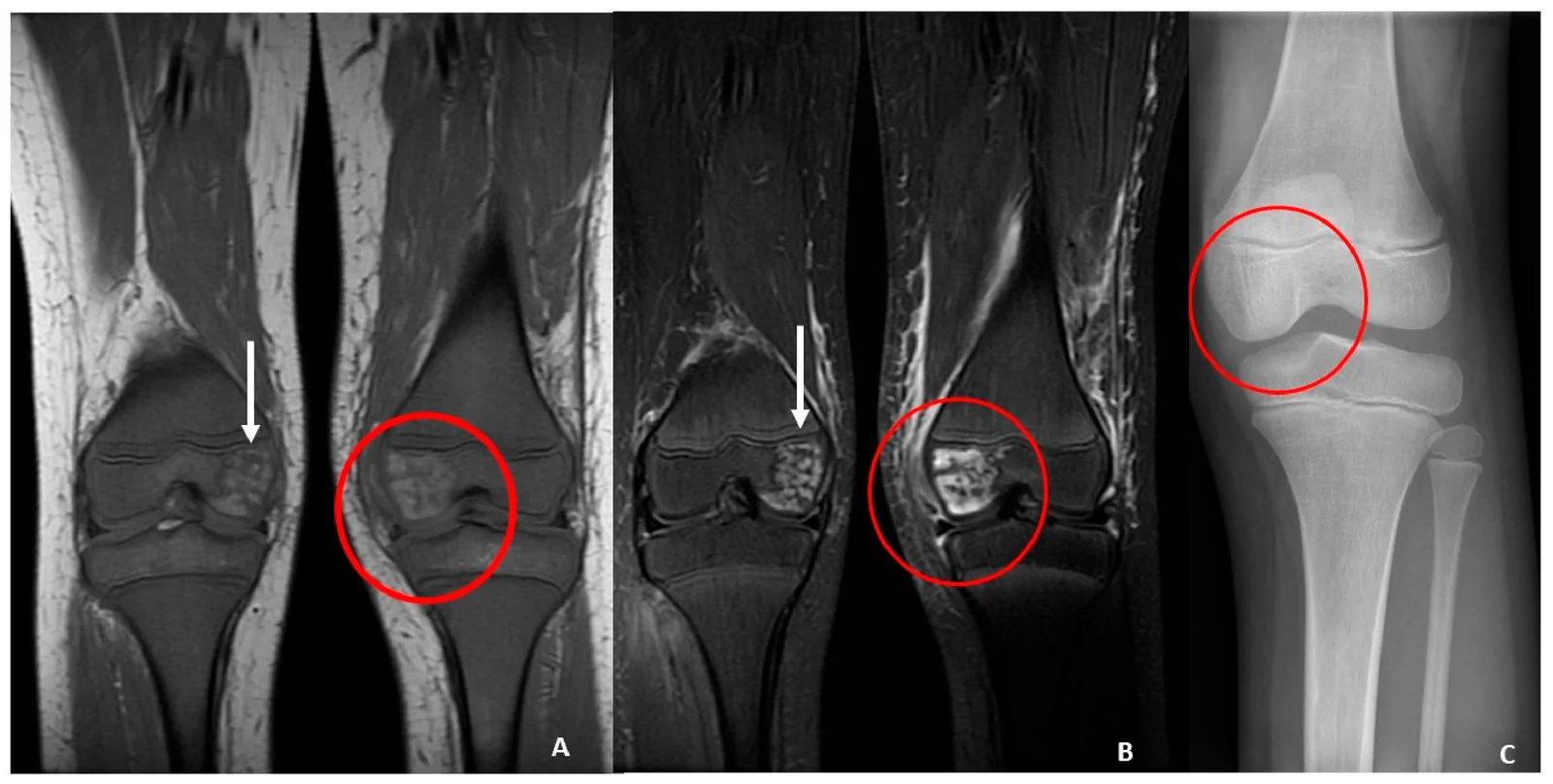
Magnetic resonance imaging (MRI) and radiographic findings of osteonecrosis in a child with sickle cell disease and left knee pain. Red circles in panels (**A**,**B**) show T1- and T2-weighted MRI images demonstrating an osteonecrotic lesion involving the left femoral medial condyle. In addition, there is a developing osteonecrotic lesion in the right femoral medial condyle (white arrow). Panel (**C**) shows the corresponding plain radiograph in the same patient, which demonstrates no visible abnormality in the left femoral medial condyle (red circle), highlighting the superior sensitivity of MRI for detecting early osteonecrosis.

**Figure 3:**
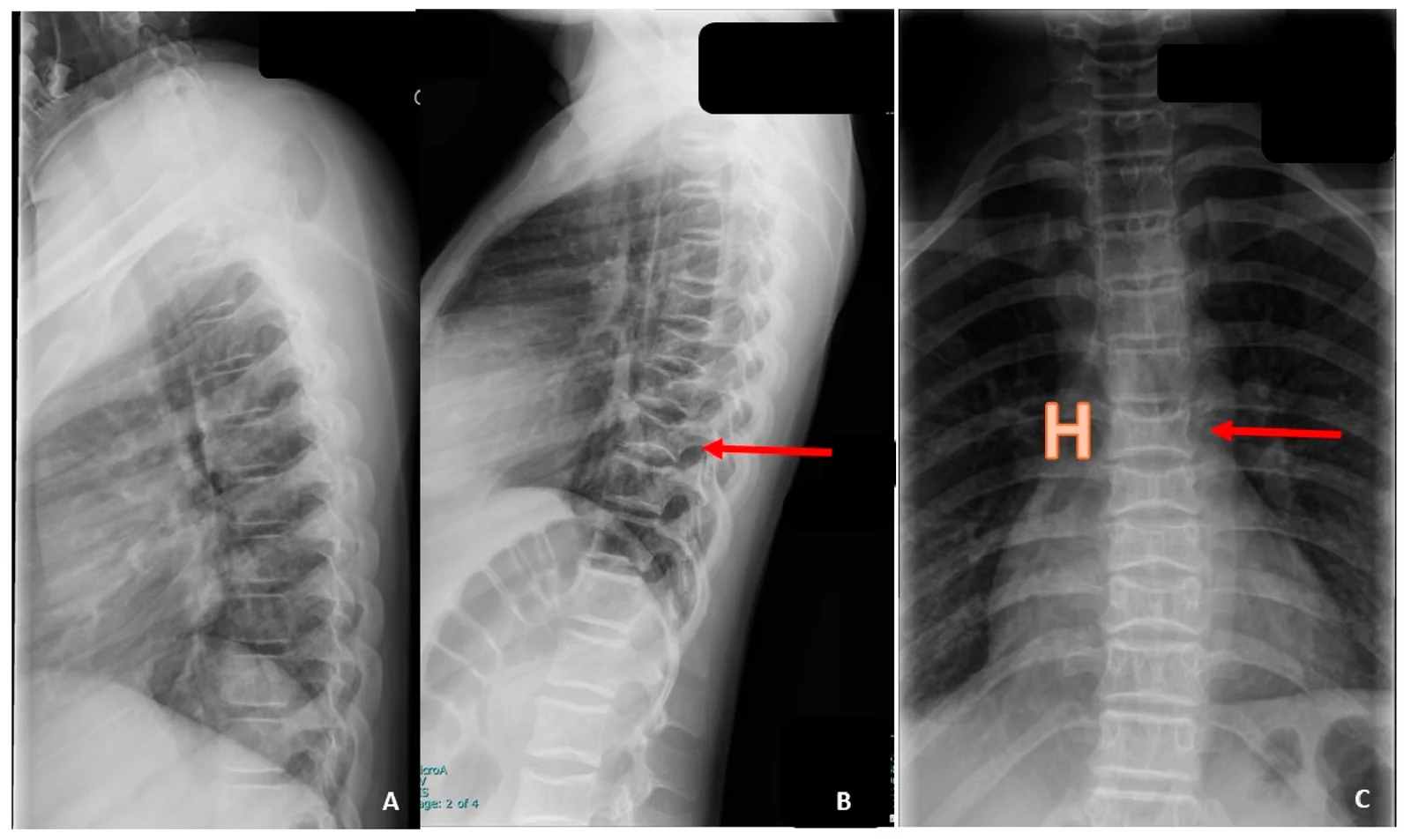
Vertebral osteonecrosis in a child with sickle cell disease. Panel (**A**) shows a normal lateral spine radiograph. Panels (**B**,**C**) show lateral and anteroposterior radiographs demonstrating the classic H-shaped vertebrae (red arrow) resulting from central endplate depression and osteonecrosis of the vertebral bodies in a 14-year-old child with sickle cell disease.

While many necrotic lesions can initially be insidious, certain exam findings can lead clinicians to suspect it. Pain and reduced range of motion, specifically loss of internal rotation at the shoulder or hip, can be early findings [19]. For ONFH, youth may present with pain referred to the groin or knee, while elbow and knee osteonecrosis may present as asymptomatic MRI lesions (Figure 2). Vertebral osteonecrosis lesions present as back pain with tenderness on palpation of the vertebral bodies (Figure 3).

## 4. Imaging Studies

Bone infarcts at the vertebral endplates present as the classic “H-shaped” vertebrae on plain X-ray. In the long bones, infarcts can initially be missed on plain film and may require MRI to visualize the bone edema, marrow changes and to distinguish between acute or chronic lesions.

**Figure 4:**
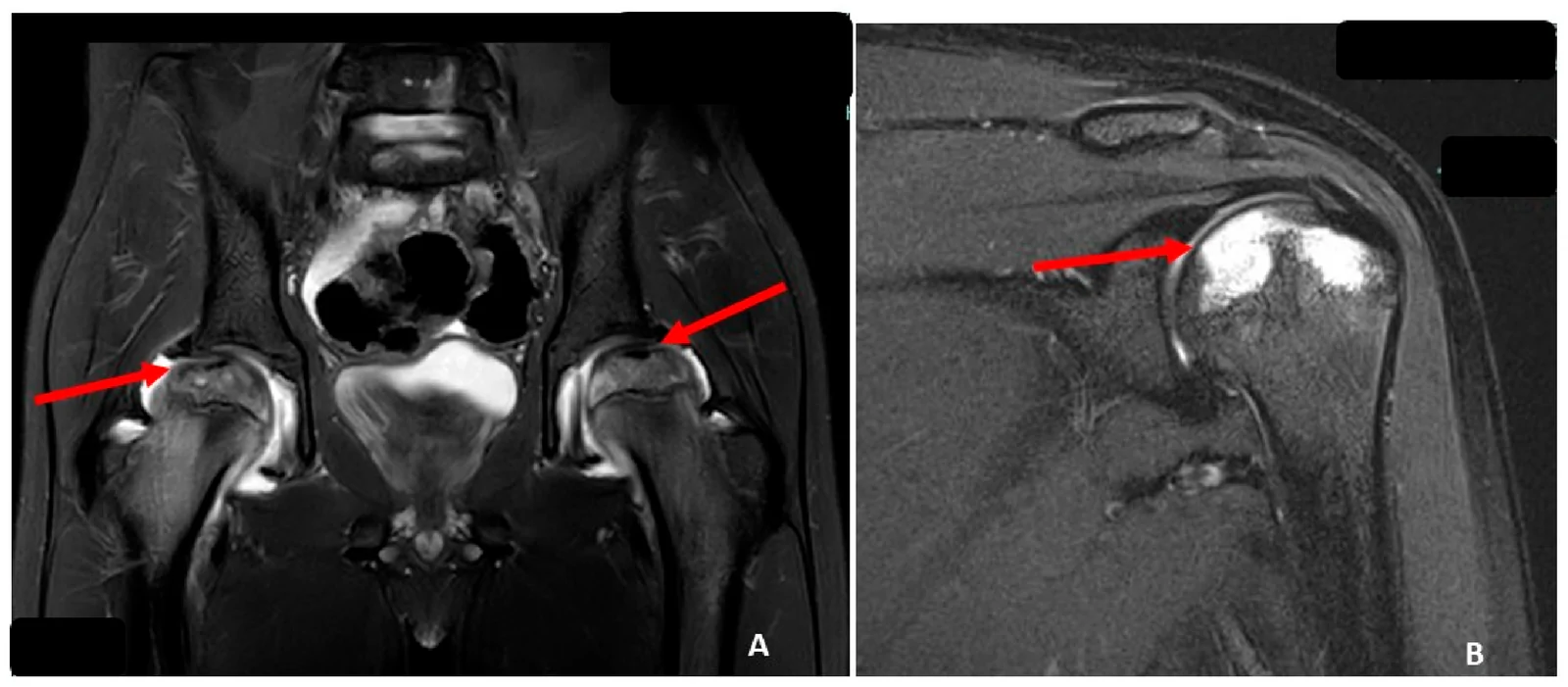
Osteonecrosis involving the articular surfaces of long bones in children with sickle cell disease. Panel (**A**) shows MRI findings of early osteonecrosis involving both femoral heads (red arrows) in a 12-year-old child. Panel (**B**) demonstrates osteonecrosis of the left humeral head (red arrow) in a 14-year-old child with sickle cell disease.

At the hip and shoulder, imaging plays a key role in the identification and staging of the lesions. Early identification and accurate staging using magnetic resonance imaging are essential because both prognosis and treatment are closely linked to disease stage. Although not specific to SCD individuals, osteonecrosis can be grossly divided into three phases: precollapse, early collapse lesions with less than 2 mm of subchondral bone depression and late collapse with >2 mm of depression and/or secondary joint changes [20]. This distinction is important because early-stage lesions may only require conservative therapy or joint-preserving strategies, whereas advanced disease with structural collapse often requires reconstructive surgery. While some expert groups recommend hip X-rays in the peripubertal years to screen for ONFH [12,18], many early lesions can only be detected by non-contrast MRI while plain radiographs may appear normal (Figure 2). In a metanalysis of 43 studies, MRI had a sensitivity of 93% and specificity of 91% for ONFH and remains the gold standard for detecting disease prior to the bone collapse phase [3]. In patients with SCD, MRI is particularly valuable because multifocal disease and early-stage lesions are common. Plain X-rays have their place as follow-up imaging to detect progression and appearance of deformity at both articular surfaces of long bones and endplate deformities following osteonecrosis in the vertebrae (Figure 4) [21,22]. Despite the usefulness of MRI, routine full body MRI screening is not currently recommended.

Although staging systems have been proposed for skeletal sites [23], most validated classification schemes have been developed for ONFH, reflecting its clinical importance and the large body of outcome data available for this location (Table 2). The Ficat–Arlet [24] and Steinberg systems [25] remain the most widely used. Originally developed for adults, the Ficat–Arlet classification, developed in 1964, categorizes disease from stage I to IV based primarily on clinical findings and radiographic changes, whereas the Steinberg system extends from stage 0 to VI and incorporates MRI findings, allowing detection and characterization of earlier disease. The Steinberg classification also accounts for lesion size, which provides important prognostic information because the hip is associated with a greater risk of femoral head collapse and progression to end-stage joint disease. The ARCO 2021 classification system [26] is a newer location-based classification for early-stage disease where collapse has not yet occurred. ARCO divides lesions in three types based on the lateral extent of the necrotic lesion related to the apex of the femoral head and acetabular edge.

Beyond staging, several imaging features provide important prognostic information. Lesion size, lesion location and the presence of subchondral collapse are among the strongest predictors of disease progression. In ONFH, larger necrotic lesions carry a substantially greater risk of collapse than smaller lesions. Necrotic involvement exceeding 30% of the femoral head is associated with progression in approximately 50% to 80% of cases, whereas lesions involving less than 5% of the femoral head progress in fewer than 30% of patients [3]. Weight-bearing involvement of the lateral femoral head and the presence of a subchondral fracture, often referred to as the “crescent sign,” are also associated with an increased likelihood of femoral head collapse and subsequent degenerative joint disease [27]. The finding marks the earliest radiographic evidence of structural failure and corresponds to Stage III of the Ficat–Arlet and Steinberg classifications [28,29].

## 5. Management

Management of osteonecrosis in children and adolescents with SCD follows a stage and location-dependent, multidisciplinary approach. Joints such as the hip and shoulder are closely followed to preserve anatomical integrity and avoid bone collapse, while other locations such as the spine are often managed symptomatically [30,31]. Early detection of osteonecrosis lesions especially in weight-bearing joints is paramount to allow for conservative management strategies to halt disease progression. The hip is the most important example as it carries the highest morbidity after joint collapse. Regardless of what stage the ONFH is diagnosed, a multidisciplinary team approach is recommended [13,32,33]. Team members should include a hematologist, physical therapist, orthopedic surgeon and metabolic bone disease specialist.

### 5.1. Conservative Management

#### 5.1.1. Analgesia and Physical Therapy

Conservative management remains the cornerstone of treatment for early-stage ONFH and plays an important role throughout the disease course. Analgesia and physical therapy are strongly recommended as first-line treatments for ONFH [31] to improve pain and function. Non-steroidal anti-inflammatory drugs are first-line choices followed by opioids for refractory cases. Given the complex pain burden experienced by many individuals with SCD, collaboration between hematology, pain medicine, rehabilitation, and orthopedic teams can help optimize a multimodal approach to symptom management.

Physical therapy is recommended for both symptomatic and asymptomatic patients, especially those with ONFH. The primary goals are to preserve joint range of motion, maintain muscle strength, reduce pain and improve functional mobility. Treatment programs commonly include strengthening of the hip abductors and extensors, gait training with or without technical aids and targeted stretching exercises [2,31,34]. Aquatic therapy can be particularly beneficial because it allows exercise with reduced joint loading while maintaining cardiovascular fitness and muscle condition. Evidence supporting physical therapy in ONFH is limited but encouraging. In the only prospective multicenter randomized trial evaluating two different treatment approaches for ONFH in SCD, Neumayr et al. assessed core decompression plus physical therapy versus physical therapy alone for early-stage (Steinberg I–III) ONFH in 38 participants with a mean age of 24 years. No statistically significant differences emerged between the groups who were followed for a mean of three years. While several limitations emerged, including small sample size, patient attrition, baseline imbalances between groups and a relatively short follow-up for a chronic condition that often progresses over decades, the study suggests that structured physical therapy may provide outcomes comparable to core decompression in the short to medium term in young adults with SCD [34].

#### 5.1.2. Bisphosphonates

Intravenous bisphosphonates can decrease bone pain and improve bone mineral density (BMD) by inhibiting osteoclast activity in the skeleton. In a multicentre review of 46 youths with SCD-related osteonecrosis who received either pamidronate, zoledronic acid or both, showed significant or complete improvements of osteonecrosis-related pain. There were no reports of vaso-occlusive crisis, stroke or other severe complications. One episode of symptomatic hypocalcemia was treated with calcium and calcitriol supplementation [7]. Although not specifically studied in SCD-related ONFH, bisphosphonates have not been shown to prevent bone collapse in other forms of non-traumatic and post-traumatic hip osteonecrosis [7,35,36,37] but a future Cochrane review is underway [38].

#### 5.1.3. Activity Modification and Weight Bearing Restriction

Activity modification and protected weight-bearing are commonly recommended for patients with symptomatic osteonecrosis, particularly during the precollapse and early collapse stages [31]. The goal is to reduce mechanical stress across the affected joint, alleviate pain, and potentially limit progression of subchondral collapse. Depending on symptom severity and lesion characteristics, temporary use of assistive devices such as crutches, canes, walkers or even wheelchairs may be considered to offload the involved extremity. However, there is limited evidence to guide the optimal degree or duration of weight-bearing restriction, and standardized protocols have not been established [30,31].

Osteonecrosis can progress despite conservative management; therefore, regular clinical follow-up and interval imaging are important, particularly in early-stage disease. Monitoring allows timely detection of further disease progression or other structural changes that may alter prognosis and prompt consideration for surgical interventions.

### 5.2. Surgical Therapy

Surgical options for ONFH in youth are limited to osteotomies and core decompression [39,40] where drilling a tract into the bone is thought to stimulate space for new blood vessel development and decrease intraosseous pressure caused by cellular pressure and inflammation. Coupling the procedure with the addition of stem cells [41,42] or bone graft [43,44] into the tract has also been described. These procedures are considered joint-sparing and are generally reserved for early-stage disease before significant structural failure has occurred. In contrast, total hip arthroplasty is typically reserved for advanced disease with femoral head collapse, secondary degenerative joint changes, or failure of previous joint-preserving interventions [39,45]. Given the complex perioperative challenges associated with SCD [46], including vaso-occlusive crises, acute chest syndrome, infection, and delayed recovery, surgical management should be undertaken by multidisciplinary teams with expertise in both SCD and complex hip reconstruction. In a large single-center study of 92 patients with a mean age of 22 years and average follow-up of 12 years demonstrated that modern cementless total hip arthroplasty provides durable pain relief and functional improvement [47]. Complications were uncommon when patients underwent multidisciplinary care and pre-operative transfusions to reduce hemoglobin S. Implant survival was excellent (94.1%) with a relatively low revision rate of 5.9% over the follow-up period [47]. Although promising, the need for future revision throughout the lifespan remains a major concern; therefore, joint preserving therapies should be exhausted prior to considering cementless total hip arthroplasty [31].

#### 5.2.1. Hyperbaric Oxygen Therapy

The evidence for hyperbaric oxygen therapy for the treatment of osteonecrosis in the context of SCD is limited to case reports and a small retrospective cohort of adult patients [48,49]. For osteonecrosis beyond the SCD, a 2021 systematic review and meta-analysis of 10 studies including 721 patients found that mostly Asian participants treated with hyperbaric oxygen therapy had 3.84 times higher odds of clinical improvement compared to controls. Currently, evidence is lacking to recommend hyperbaric oxygen therapy as standard treatment for osteonecrosis in SCD youth [17,31].

#### 5.2.2. Vertebroplasty

At the current time, there is no evidence to support vertebroplasty in youths with SCD who sustain vertebral endplate infarcts leading to deformity.

## 6. Other Important Elements of Bone Health

Optimizing bone health is an important component of osteonecrosis management in SCD. Given the high prevalence of metabolic and endocrine abnormalities in SCD, assessment of vitamin D status, pubertal development, gonadal function, thyroid function, and BMD should be considered as part of a comprehensive approach to skeletal health. Delayed pubertal growth and lower peak bone mass may also help explain the high fracture burden in adolescents with sickle cell disease [50]. Endocrine complications are highly prevalent in individuals with SCD. A cross-sectional study involving 52 Italian pediatric patients reported endocrine abnormalities in 92% of participants, with vitamin D deficiency representing the most common finding [51]. These disorders may adversely affect bone accrual during childhood and contribute to progressive skeletal fragility over time. Consistent with this, longitudinal data have identified increasing age, male sex, hypothyroidism, and hypogonadism as independent predictors of fracture risk [52]. Patients with hypogonadism appear particularly vulnerable, demonstrating a higher risk of fractures in association with low BMD and vitamin D deficiency despite ongoing transfusion and iron chelation therapy [52].

### Why Children Are Different

Osteonecrosis in children with SCD differs from that in adults in several clinically important ways. Although the underlying pathology is similar, pediatric patients are more likely to harbor asymptomatic lesions, making diagnosis based on clinical symptoms alone unreliable. Consequently, clinicians should maintain a high index of suspicion and have a low threshold for obtaining advanced imaging such as an MRI when osteonecrosis is suspected but plain X-rays are normal. Secondly, skeletally immature children retain the capacity for bone remodeling and growth. Reports of vertebral body [7] and femoral head [53] reshaping in children with SCD suggest that growth and bone maturation may allow partial restoration of bone architecture in certain individuals who have not yet reached final height. This remodeling potential, which is largely absent in adults whose growth plates have fused, has important implications for prognosis, surveillance and treatment decisions.

## 7. Conclusions

As advances in newborn screening, supportive care, and disease-modifying therapies continue to improve survival, the long-term musculoskeletal consequences of SCD are becoming increasingly apparent. Among these, osteonecrosis remains one of the most common and disabling complications, often affecting children, adolescents, and young adults during critical periods of growth, physical and social development.

Despite its clinical importance, osteonecrosis remains challenging to prevent and treat. Early symptoms may be subtle, and structural joint damage can progress before radiographic changes become evident. Timely recognition, appropriate imaging, and early referral to multidisciplinary teams are therefore essential. In addition to addressing pain and preserving joint function, comprehensive management should include optimization of bone health and treatment of underlying endocrine and metabolic abnormalities that may contribute to skeletal fragility.

Important knowledge gaps remain regarding the natural history of osteonecrosis in SCD, especially in locations beyond the hip. Future research should focus on validated risk prediction tools, exploring whether bisphosphonate therapy can improve joint surface reshaping and assessing which surgical interventions, if any, can preserve joint integrity before irreversible collapse occurs.

## Figures and Tables

**Table 1:** Site-specific clinical features and screening recommendations for osteonecrosis in youth with Sickle Cell Disease.

Site	Important Features	Screening Recommendations
Femoral head	Most common site for symptomatic osteonecrosis; 70% of cases are bilateral, incidence increases with age, seen as early as age 6 years.	Should be systematically screened beginning at age 5–12 years or earlier if pain or clinical findings on exam
Humeral head	Often asymptomatic, 33% bilateral lesions; seen as early as age 5 years.	Screen if clinical suspicion
Vertebrae	H-shaped deformity from endplate infarctions; can be asymptomatic or symptomatic.	Screen if back pain is present
Knees and elbows	Less common, often asymptomatic, phenotype ill-defined.	Screening if clinical suspicion

**Table 2:** Comparison of the Ficat and Arlet, Steinberg, and ARCO Classification Systems for Osteonecrosis of the Femoral Head.

Features	Ficat and Arlet Classification	Steinberg Classification	ARCO Classification (Association Research Circulation Osseous)
**Primary basis**	Clinical findings and plain radiographs	Radiographs, CT/MRI and lesion extent	Radiographs, MRI, and CT using standardized staging
**Number of stages**	I–IV	0–VI	I–IV
**Early disease detection**	Limited; Stage I requires normal radiographs with abnormal MRI or bone scan	Excellent; includes Stage 0 (preclinical) and Stage I (MRI changes only)	Excellent; MRI is central for early diagnosis
**Assessment of lesion size**	No	Yes; lesions categorized by femoral head involvement (<15%, 15–30%, >30%)	Yes; incorporates lesion size and location, particularly before collapse
**Assessment of lesion location**	No	Limited	Yes; emphasizes involvement of the weight-bearing surface
**Stage I**	Normal radiographs with abnormal MRI or bone scan	Stage 0: Normal radiographs, MRI and bone scan Stage I: Normal radiographs with abnormal MRI or bone scan	MRI abnormalities with normal radiographs
**Stage II**	Sclerosis and/or cystic changes without femoral head collapse	Sclerosis and/or cystic changes without collapse or flattening	Radiographic abnormalities without subchondral fracture or femoral head flattening
**Stage III**	Crescent sign indicating subchondral collapse without joint-space narrowing	Crescent sign with early collapse but no femoral head flattening	Subchondral fracture (crescent sign) with preserved joint space. Stage IIIA: Femoral head depression ≤2 mm. Stage IIIB: Femoral head depression >2 mm.
**Stage IV**	Femoral head flattening with secondary osteoarthritis	Femoral head flattening	Femoral head collapse and/or flattening
**Advanced arthritis**	Included within Stage IV	Stage V: Joint-space narrowing Stage VI: Advanced degenerative arthritis	Secondary osteoarthritis incorporated into Stage IV

## Data Availability

No new data were created or analyzed in this study. Data sharing is not applicable to this article.

## References

[1] Thomson A.M., A McHugh T., Oron A.P., Teply C., Lonberg N., Tella V.V., Wilner L.B., Fuller K., Hagins H., Aboagye R.G. (2023). Global, regional, and national prevalence and mortality burden of sickle cell disease, 2000–2021: A systematic analysis from the Global Burden of Disease Study 2021. Lancet Haematol..

[2] Mulyana A.M., Rakhmawati W., Pramukti I., Lukman M., Wartakusumah R., Mediani H.S. (2024). Bone Disease among Children with Sickle Cell Disease: A Scoping Review of Incidence and Interventions. J. Multidiscip. Healthc..

[3] Ha A.S., Chang E.Y., Bartolotta R.J., Bucknor M.D., Chen K.C., Ellis H.B., Flug J., Leschied J.R., Ross A.B., Sharma A. (2022). ACR Appropriateness Criteria^®^ Osteonecrosis: 2022 Update. J. Am. Coll. Radiol..

[4] Cheng E.Y., Mirzaei A., Sugano N., Hernigou P., Jones L.C., Koo K.H., Drescher W.R., Cui Q., Sierra R.J., Goodman S.B. (2025). Osteonecrosis: A More Appropriate Term than Avascular Necrosis-Pathophysiologic Rationale. J. Arthroplast..

[5] Adekile A.D., Gupta R., Al-Khayat A., Mohammed A., Atyani S., Thomas D. (2019). Risk of avascular necrosis of the femoral head in children with sickle cell disease on hydroxyurea: MRI evaluation. Pediatr. Blood Cancer.

[6] Adesina O., Brunson A., Keegan T.H.M., Wun T. (2017). Osteonecrosis of the femoral head in sickle cell disease: Prevalence, comorbidities, and surgical outcomes in California. Blood Adv..

[7] Grimbly C., Escagedo P.D., Jaremko J.L., Bruce A., Alos N., Robinson M.E., Konji V.N., Page M., Scharke M., Simpson E. (2022). Sickle cell bone disease and response to intravenous bisphosphonates in children. Osteoporos. Int..

[8] Milner P.F., Kraus A.P., Sebes J.I., Sleeper L.A., Dukes K.A., Embury S.H., Bellevue R., Koshy M., Moohr J.W., Smith J. (1993). Osteonecrosis of the humeral head in sickle cell disease. Clin. Orthop. Relat. Res..

[9] Sadat-Ali M., Ammar A., Corea J.R., Ibrahim A.W. (1994). The spine in sickle cell disease. Int. Orthop..

[10] Mesleh Shayeb A., Smeltzer M.P., Kaste S.C., Brown A., Estepp J.H., Nottage K.A. (2018). Vaso-occlusive crisis as a predictor of symptomatic avascular necrosis in children with sickle cell disease. Pediatr. Blood Cancer.

[11] Milner P.F., Kraus A.P., Sebes J.I., Sleeper L.A., Dukes K.A., Embury S.H., Bellevue R., Koshy M., Moohr J.W., Smith J. (1991). Sickle cell disease as a cause of osteonecrosis of the femoral head. N. Engl. J. Med..

[12] Voi V., Turrini S., Mattavelli M., Vigliani V., Margarita G., Ferrero G.B. (2023). Occult ischemic bone lesions in children with sickle cell disease: A study of prevalence. Eur. J. Haematol..

[13] Ouederni M., Rouag H., Ben Fraj I., Rekaya S., Kouki R., Lamouchi T., Zaiter I., Mellouli F., Bejaoui M., Ben Khaled M. (2023). Incidence and risk factors for osteonecrosis of the femoral head in five hundred and ten sickle cell disease paediatric patients. Int. Orthop..

[14] Worrall D., Smith-Whitley K., Wells L. (2016). Hemoglobin to Hematocrit Ratio: The Strongest Predictor of Femoral Head Osteonecrosis in Children with Sickle Cell Disease. J. Pediatr. Orthop..

[15] Kirkham J.K., Estepp J.H., Weiss M.J., Rashkin S.R. (2023). Genetic Variation and Sickle Cell Disease Severity: A Systematic Review and Meta-Analysis. JAMA Netw. Open.

[16] Daltro P.B., Ribeiro T.O., Daltro G.C., Meyer R.J., Fortuna V. (2020). CD4^+^ T Cell Profile and Activation Response in Sickle Cell Disease Patients with Osteonecrosis. Mediat. Inflamm..

[17] Kavanagh P.L., Fasipe T.A., Wun T. (2022). Sickle Cell Disease: A Review. JAMA.

[18] Yates A.M., Aygun B., Nuss R., Rogers Z.R. (2024). Health Supervision for Children and Adolescents With Sickle Cell Disease: Clinical Report. Pediatrics.

[19] Mankin H.J. (1992). Nontraumatic necrosis of bone (osteonecrosis). N. Engl. J. Med..

[20] Bar M., Ott S.M., Lewiecki E.M., Sarafoglou K., Wu J.Y., Thompson M.J., Vaux J.J., Dean D.R., Saag K.G., Hashmi S.K. (2020). Bone Health Management After Hematopoietic Cell Transplantation: An Expert Panel Opinion from the American Society for Transplantation and Cellular Therapy. Biol. Blood Marrow Transplant..

[21] Bedair E., Almaslamani N., Yassin M. (2023). Radiological manifestation of avascular necrosis (AVN) in sickle cell disease (SCD): A review of diagnostic imaging. Acta Biomed..

[22] Ejindu V.C., Hine A.L., Mashayekhi M., Shorvon P.J., Misra R.R. (2007). Musculoskeletal manifestations of sickle cell disease. Radiographics.

[23] Formica M., Zanirato A., Cavagnaro L., Basso M., Divano S., Lamartina C., Berjano P., Felli L., Formica C. (2018). Vertebral body osteonecrosis: Proposal of a treatment-oriented classification system. Eur. Spine J..

[24] Mont M.A., Marulanda G.A., Jones L.C., Saleh K.J., Gordon N., Hungerford D.S., Steinberg M.E. (2006). Systematic analysis of classification systems for osteonecrosis of the femoral head. J. Bone Jt. Surg. Am..

[25] Steinberg M.E., Hayken G.D., Steinberg D.R. (1995). A quantitative system for staging avascular necrosis. J. Bone Jt. Surg. Br..

[26] Koo K.H., Mont M.A., Cui Q., Hines J.T., Yoon B.H., Novicoff W.M., Lee Y.J., Cheng E.Y., Drescher W., Hernigou P. (2022). The 2021 Association Research Circulation Osseous Classification for Early-Stage Osteonecrosis of the Femoral Head to Computed Tomography-Based Study. J. Arthroplast..

[27] Kenzora J.E., Glimcher M.J. (1985). Pathogenesis of idiopathic osteonecrosis: The ubiquitous crescent sign. Orthop. Clin. N. Am..

[28] Sugano N., Atsumi T., Ohzono K., Kubo T., Hotokebuchi T., Takaoka K. (2002). The 2001 revised criteria for diagnosis, classification, and staging of idiopathic osteonecrosis of the femoral head. J. Orthop. Sci..

[29] Plakseychuk A.Y., Shah M., Varitimidis S.E., Rubash H.E., Sotereanos D. (2001). Classification of osteonecrosis of the femoral head. Reliability, reproducibility, and prognostic value. Clin. Orthop. Relat. Res..

[30] Martí-Carvajal A.J., Solà I., Agreda-Pérez L.H. (2019). Treatment for avascular necrosis of bone in people with sickle cell disease. Cochrane Database Syst. Rev..

[31] Yawn B.P., Buchanan G.R., Afenyi-Annan A.N., Ballas S.K., Hassell K.L., James A.H., Jordan L., Lanzkron S.M., Lottenberg R., Savage W.J. (2014). Management of sickle cell disease: Summary of the 2014 evidence-based report by expert panel members. JAMA.

[32] Simon A.L., Montanari L., Mallet C., Ilharreborde B. (2026). Orthopedic complications of sickle-cell disease in children. Orthop. Traumatol. Surg. Res..

[33] Gupta P., Shrivastava S., Kumar R. (2025). Musculoskeletal complications in sickle cell disease: Pathophysiology, diagnosis and management. Best. Pr. Res. Clin. Rheumatol..

[34] Neumayr L.D., Aguilar C., Earles A.N., Jergesen H.E., Haberkern C.M., Kammen B.F., Nancarrow P.A., Padua E., Milet M., Stulberg B.N. (2006). Physical therapy alone compared with core decompression and physical therapy for femoral head osteonecrosis in sickle cell disease. Results of a multicenter study at a mean of three years after treatment. J. Bone Jt. Surg. Am..

[35] Lee Y.K., Ha Y.C., Cho Y.J., Suh K.T., Kim S.Y., Won Y.Y., Min B.W., Yoon T.R., Kim H.J., Koo K.H. (2015). Does Zoledronate Prevent Femoral Head Collapse from Osteonecrosis? A Prospective, Randomized, Open-Label, Multicenter Study. J. Bone Jt. Surg. Am..

[36] Ramachandran M., Ward K., Brown R.R., Munns C.F., Cowell C.T., Little D.G. (2007). Intravenous bisphosphonate therapy for traumatic osteonecrosis of the femoral head in adolescents. J. Bone Jt. Surg. Am..

[37] Padhye B., Dalla-Pozza L., Little D.G., Munns C.F. (2013). Use of zoledronic acid for treatment of chemotherapy related osteonecrosis in children and adolescents: A retrospective analysis. Pediatr. Blood Cancer.

[38] Ball S., Thomas R., Galloway A.M., Davies L.M., Gray K., Brennan S., Johnston R.V., Williams N., Munns C.F., Pacey V. (2026). Treatment for primary avascular necrosis of the lower limb in childhood. Cochrane Database Syst. Rev..

[39] Kamath A.F., McGraw M.H., Israelite C.L. (2015). Surgical management of osteonecrosis of the femoral head in patients with sickle cell disease. World J. Orthop..

[40] Griffith M.S., Shaw K.A., Hattaway J.K., Schrader T. (2021). Core Decompression and Bone Marrow Aspirate Concentrate in the Treatment of Femoral Head Avascular Necrosis in Pediatric Sickle Cell Disease: Can We Improve Natural History?. J. Pediatr. Orthop..

[41] Baghdadi S., Chern I., Hanstein R., Mehraban Alvandi L., Fornari E. (2023). Femoral Head Core Decompression and Bone Marrow Concentrate Injection in Pediatric Sickle-cell Related Avascular Necrosis. J. Pediatr. Orthop..

[42] Gangji V., Hauzeur J.P., Matos C., De Maertelaer V., Toungouz M., Lambermont M. (2004). Treatment of osteonecrosis of the femoral head with implantation of autologous bone-marrow cells. A pilot study. J. Bone Jt. Surg. Am..

[43] Steinberg M.E., Larcom P.G., Strafford B., Hosick W.B., Corces A., Bands R.E., Hartman K.E. (2001). Core decompression with bone grafting for osteonecrosis of the femoral head. Clin. Orthop. Relat. Res..

[44] Keizer S.B., Kock N.B., Dijkstra P.D., Taminiau A.H., Nelissen R.G. (2006). Treatment of avascular necrosis of the hip by a non-vascularised cortical graft. J. Bone Jt. Surg. Br..

[45] van Kouswijk H.W., Bus M.P.A., Gademan M.G.J., Nelissen R., de Witte P.B. (2025). Total Hip Arthroplasty in Children: A Dutch Arthroplasty Register Study with Data from 283 Hips. J. Bone Jt. Surg. Am..

[46] Alfaya F.F., Ghazy R.M., Hammouda E.A., Mahfouz A.A., Faya H.K., Asiri M.A.M., Alalmaie O.H.M., Alshahrani N.Y., Alqahtani A.Z.A., Alshahrani A.Y. (2024). Total Hip Arthroplasty Complications in Sickle Cell Disease: Systematic Review and Meta-Analysis. J. Clin. Med..

[47] AlOmran A.S. (2023). Survival of total hip arthroplasty (THA) in sickle cell disease. Arch. Orthop. Trauma. Surg..

[48] Alshurafa A., Elhissi M., Yassin M.A. (2022). Complete resolution of stage II avascular necrosis affecting three joints by hyperbaric oxygen in a patient with sickle cell disease: A case report. Front. Med..

[49] Alshurafa A., Soliman A.T., De Sanctis V., Ismail O., Abu-Tineh M., Hemadneh M.K.E., Rashid F.R., Yassin K., Qasim H., Nashwan A.J. (2023). Clinical and epidemiological features and therapeutic options of avascular necrosis in patients with sickle cell disease (SCD): A cross-sectional study. Acta Bio Medica Atenei Parm..

[50] Fung E.B., Kawchak D.A., Zemel B.S., Rovner A.J., Ohene-Frempong K., Stallings V.A. (2008). Markers of bone turnover are associated with growth and development in young subjects with sickle cell anemia. Pediatr. Blood Cancer.

[51] Mandese V., Bigi E., Bruzzi P., Palazzi G., Predieri B., Lucaccioni L., Cellini M., Iughetti L. (2019). Endocrine and metabolic complications in children and adolescents with Sickle Cell Disease: An Italian cohort study. BMC Pediatr..

[52] Fung E.B., Sarsour I., Manzo R., Lal A. (2025). Bone quality is associated with fragility fracture in patients with hemoglobinopathies. J. Clin. Densitom..

[53] Itzep N.P., Jadhav S.P., Kanne C.K., Sheehan V.A. (2019). Spontaneous healing of avascular necrosis of the femoral head in sickle cell disease. Am. J. Hematol..

